# Inverted left atrial appendage mimicking potential left atrial thrombus in an infant following cardiac surgery: a case report

**DOI:** 10.1093/ehjcr/ytae105

**Published:** 2024-03-14

**Authors:** Muhammad Najih Liaqath Ali, Tara Bharucha, Markku Kaarne, Nicholas Hayes

**Affiliations:** 1 Department of Paediatric Cardiology, University Hospital of Southampton NHS Trust, Tremona Road, Southampton, Hampshire SO16 6YD, UK; 1 Department of Paediatric Cardiology, University Hospital of Southampton NHS Trust, Tremona Road, Southampton, Hampshire SO16 6YD, UK; Department of Congenital Cardiac Surgery, University Hospital of Southampton NHS Trust, Southampton, UK; 1 Department of Paediatric Cardiology, University Hospital of Southampton NHS Trust, Tremona Road, Southampton, Hampshire SO16 6YD, UK

**Keywords:** Left atrium, Intracardiac mass, Appendage, Intussusception, Case report

## Abstract

**Background:**

Inverted left atrial appendage (iLAA) is an infrequent complication following cardiac surgery, seen both in children and adults. Following a recent encounter, this review article is aimed to remind the reader about its occurrence, clinical manifestations, differential diagnoses, and management options.

**Case summary:**

A 3-month-old baby underwent successful surgical repair of a large ventricular septal defect. Intraoperative epicardial echocardiogram at the end of the case demonstrated an unexpected left atrial mass, raising suspicion of a thrombus adjacent to the mitral valve. Urgent re-establishment of cardiopulmonary bypass and exploration of the left atrium did not reveal a thrombus, but this was subsequently identified as an iLAA. This completely resolved after manual external reduction of the appendage.

**Discussion:**

Published literature is confined to case reports only, with most cases observed post-operatively, but some occurring spontaneously. Awareness of this unusual manifestation is particularly important in the intraoperative period as it can usually be addressed without the need for further cardiopulmonary bypass.

**Learning points**

- Awareness of existence of inverted left atrial appendage as a post-operative entity.
- Routine post bypass echocardiographic as well as surgical inspection of atriums is good practice.
- Post-operative atrial mass should raise the suspicion of appendiceal inversion, and early intraoperative recognition could avoid the need for re-intervention.

## Introduction

A hyperechoic intracardiac mass (especially after cardiac surgery) is usually considered to be a thrombus, vegetation, or rarely a tumour.^1^ Inverted left atrial appendage (iLAA) is an infrequent complication following cardiac surgery^2^ and rarely occurs spontaneously.^3–5^ We report an interesting, yet fairly typical presentation, where a concerning intraoperative epicardial echocardiogram led to re-exploration of the left atrium to exclude thrombus. This case report aims to remind the reader of the occurrence, clinical manifestations, differential diagnoses, and management options of iLAA.

## Summary figure

**Figure:**
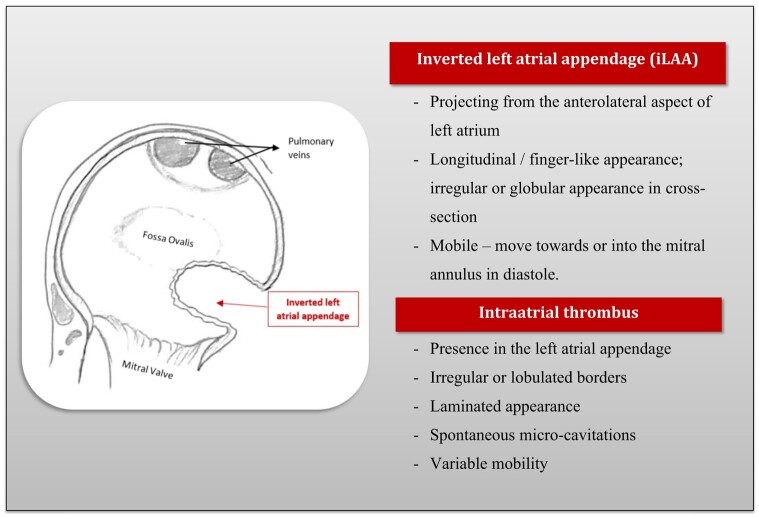


## Case summary

A 3-month-old male baby presented with failure to thrive and a cardiac murmur. Echocardiogram demonstrated an isolated large 6 mm × 9 mm perimembranous ventricular septal defect (VSD) with unrestrictive left to right shunt. This was causing pulmonary plethora, congestive cardiac failure, and significant left heart volume overload. There were no additional intra/extracardiac anomalies. Despite maximal medical therapy, he remained symptomatic and was accepted for surgical VSD closure.

He underwent VSD closure with porcine pericardial patch via a right atrial approach utilizing standard heparinization, cold blood cardioplegia, cardiopulmonary bypass (CPB), and aortic cross clamp (AXC). The surgical procedure had CPB duration of 47 min and AXC time of 35 min. Cardiac rhythm, biochemical, and haemodynamic parameters remained stable throughout.

**Figure 1:**
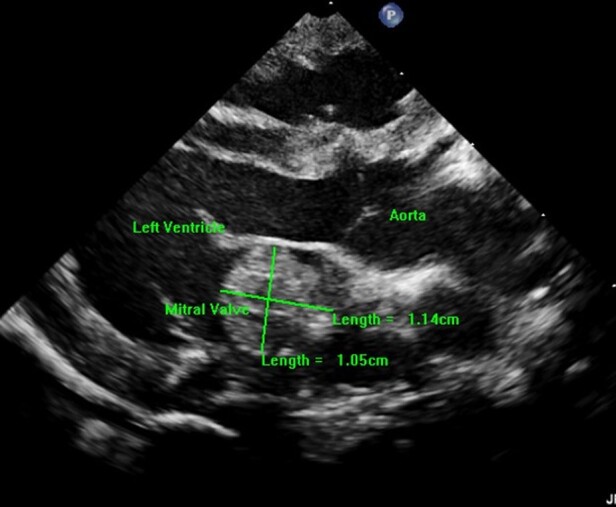
Epicardial long-axis view: mass-like appearance of the inverted left atrial appendage measuring 10 mm × 11 mm as it protrudes into the left atrium (LA). Note its position between the left pulmonary vein and the insertion of the mitral valve at the atrioventricular groove (arrow).

**Figure 2:**
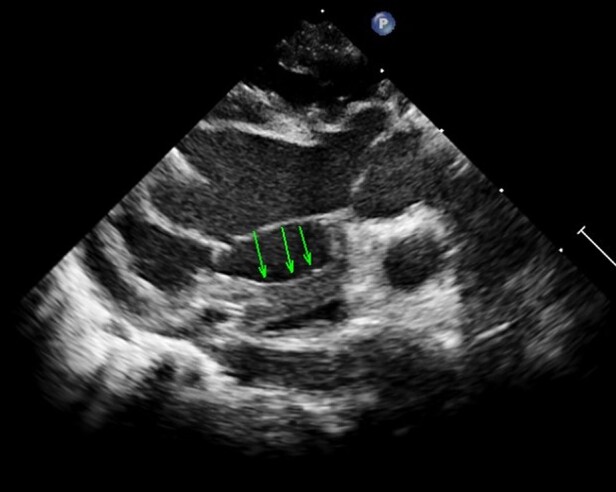
Epicardial long-axis modified view: finger-like or tongue-like extension of the echogenic structure (arrows) arising from the left atrial free wall, hindering the movement of mitral leaflet.

After separation from CPB, routine epicardial echocardiogram showed no residual VSD and good ventricular function, but an unexpected left atrial ‘mass’ was identified. This was a single well circumscribed, globular homogenously hyperechogenic mass within the left atrium with a smooth edge. On multiple planes, the mobile mass measured 10 mm × 11 mm, with a tongue- or finger-like appearance, without a peduncle, extending from the free wall of the left atrium. With each cardiac cycle, the ‘mass’ protruded closer to the mitral annulus in diastole (*Figure 1*, Supplementary material online, *Video S1*). On colour Doppler interrogation, no obstruction to pulmonary veins or mitral inflow was noted. This was confirmed to be above the mitral valve and away from the interatrial septum on short-axis interrogation (*Figure 2*, Supplementary material online, *Video 2*). An intracardiac thrombus was the primary differential diagnosis; however, he was adequately anticoagulated, the activated coagulation time was maintained in the desired range throughout, and no thrombi were observed in the CPB circuit.

As potential embolization of large left atrial thrombus can have devastating consequences, the decision was made to urgently explore the left atrium and evacuate the thrombus. Cardiopulmonary bypass was reinstituted, the aorta cross-clamped, and the heart arrested with cold blood cardioplegia. The right atriotomy was re-opened, and the left atrium was inspected through the atrial septum. Surprisingly, no thrombus or obvious pathology was identified. The child was weaned from CPB on stable haemodynamic parameters. Total CPB time of 47 + 29 = 76 min and AXC time of 35 + 21 = 56 min.

**Figure 3:**
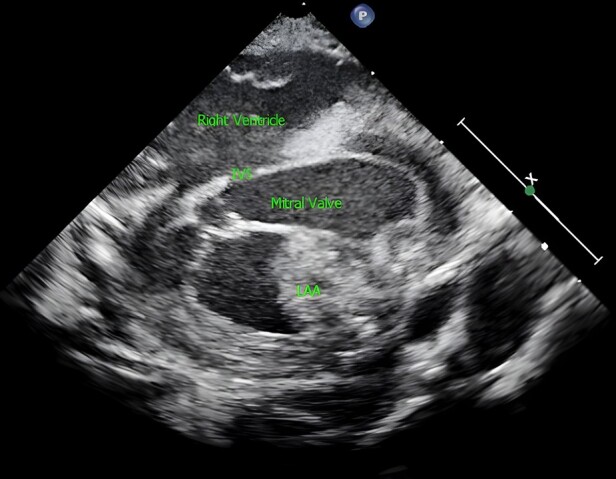
Epicardial short-axis view: globular smooth-edged structure (LAA) seen just above the mitral valve, away from the interventricular septum (IVS), impinging on to the mitral annulus.

Yet, repeat epicardial echocardiogram continued to show persistence of the mass, unchanged from previous appearances (*Figure 3*, Supplementary material online, *Video 3*). It was at this point consideration was given to a potential iLAA, which was everted externally using a gentle suction catheter. This resulted in complete resolution of the ‘mass’ on the repeat epicardial echocardiogram.

The baby recovered well following the surgery, extubated within 24 h, and had an uneventful recovery with no recurrence of appendiceal inversion. He is currently thriving well without any cardiovascular concerns.

## Discussion

Only 34 cases have been reported hitherto, with patients aged 7 days to 77 years. Various pathophysiologic theories have been proposed including spontaneous inversion, negative suction through the intracardiac vents during de-airing manouevres,^6,7^ external compression from lung hyperinflation, and iatrogenic inadvertent inclusion of the LAA while closing the superior end of VSDs.^8^

**Figure 4:**
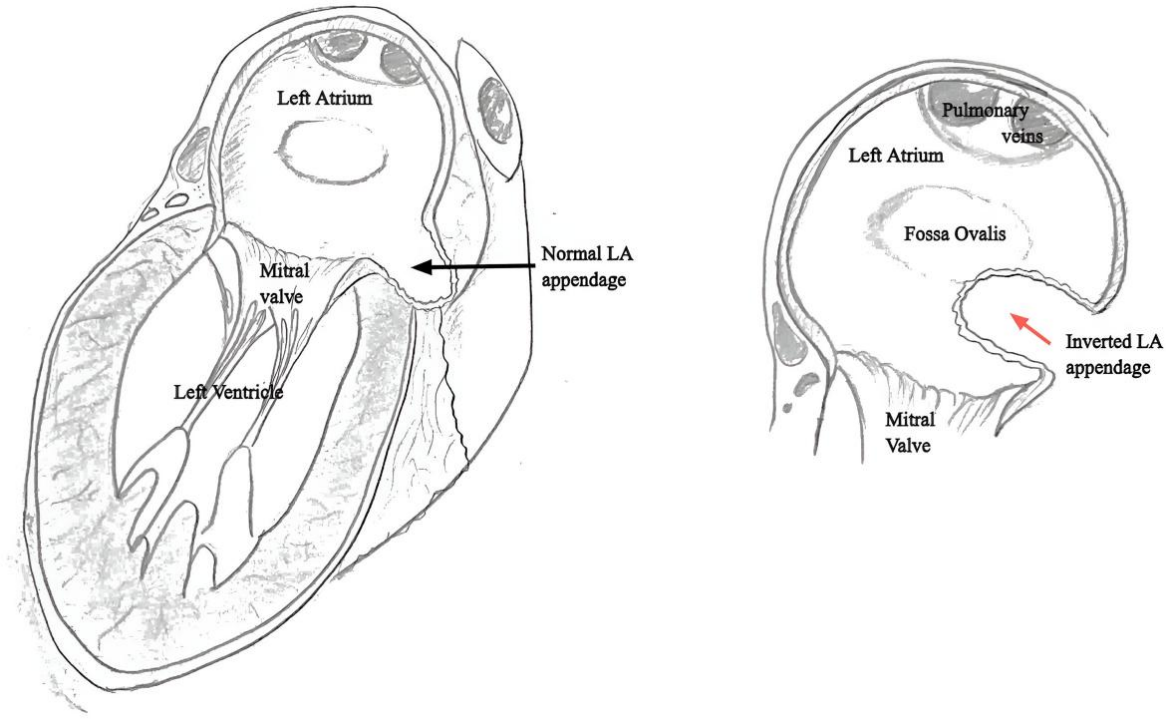
Illustrative image of LAA inversion: (left) a normal apical two-chamber view of the left atrium and left ventricle with a normal left atrial (LA) appendage. (Right) Zoomed view of the left atrial appendage involuting inside the left atrial cavity from the free wall.

Transthoracic echocardiography can have limitations in assessing the atrial appendages, hence transoesophageal echocardiography (TOE) is preferred for full assessment. Aronson *et al*.^9^ described the echocardiographic features of iLAA. It will be: mobile, projecting from the anterolateral aspect of left atrium, move towards or into the mitral annulus in diastole, have a longitudinal appearance of finger-like projection (highly suggestive), and appear irregular or globular in cross-section (*Figure 4*).^9^ In short-axis TOE, the mass has a typical location just superior to the mitral valve (MV) and inferior to the pulmonary veins.^3^

Although it has been presumed to be benign in nature due to its endothelial lining and probable low likelihood of thrombus,^10^ cases of haemorrhagic transformation,^11^ adhesion,^12^ and incarceration with impending appendiceal necrosis^13,14^ have been reported. It can also cause haemodynamic consequence due to a mass effect inside the left atrium, by obstructing the mitral inflow^3^ or the pulmonary venous inflow, as well as causing arrhythmias.^13^

The majority of reported cases spontaneously reverts once the heart is filled, but if not, iLAA should be addressed to prevent rare, but serious complications requiring surgical re-intervention.^6^ Manual manipulation is the usual mode of eversion. The chance of further spontaneous inversion is low but heightened when the neck/base of the left atrial appendage (LAA) is particularly narrow,^7^ and complete ligation of LAA may be considered to prevent recurrence.

The key for diagnosis is the primary awareness of its existence and ruling out other differential diagnoses strategically. Intraoperative surgical assessment can confirm the diagnosis and permit external digital eversion or forceps/suction catheter retraction without the need for additional CPB.^15^ If this fails, internal eversion, purse-string exclusion of LAA with/without ligation can be necessary. When identified late post-operatively, redo-sternotomy may be considered after detailed assessment of the appendage for possible necrosis and mitral obstruction.^3^

## Conclusion

Inverted LAA is a rarely seen complication of cardiac surgery, occurring in any age group. Intraoperative identification, either by surgical inspection or echocardiographic visualization, allows immediate remediation and precludes avoidable re-operation.

## Supplementary Material

ytae105_Supplementary_Data

## Data Availability

The data underlying this article will be shared on reasonable request to the corresponding author.

## Lead author biography

Muhammad Najih L. is a paediatric cardiology trainee at the University Hospital of Southampton NHS Trust, UK, with a special interest in advanced echocardiography and cross-sectional cardiac imaging.

## Supplementary material

**Consent:** The authors confirm that written consent for the submission and publication of this case, including echocardiographic images, has been obtained from the patient’s parents in line with COPE guidance

**Funding:** None declared.
